# Nucleation Mechanisms of CO_2_ Hydrate Reflected by Gas Solubility

**DOI:** 10.1038/s41598-018-28555-y

**Published:** 2018-07-11

**Authors:** Peng Zhang, Qingbai Wu, Cuicui Mu, Xueping Chen

**Affiliations:** 1 State Key Laboratory of Frozen Soil Engineering, Northwest Institute of Eco-Environment and Resources, Chinese Academy of Sciences, Lanzhou, 730000 China; 2 Key Laboratory of Western China’s Environmental Systems (Ministry of Education), College of Earth and Environmental Sciences, Lanzhou University, Lanzhou, 730000 China

## Abstract

The concentration of gas has been confirmed as a key factor dominating hydrate nucleation. In this study, CO_2_ hydrates were formed in pure water and a sodium dodecyl sulphate (SDS) solution using a temperature reduction method under constant pressure at different temperatures. The dissolving properties of CO_2_ throughout the whole induction period were investigated in detail. The experimental results showed that the ‘memory effect’ of hydrate might not be attributed to residual water structures after hydrate dissociation. Instead, residual gas molecules in the aqueous phase should receive more attention. Hydrate nucleation was confirmed to be a type of chain reaction. Low temperature was a significant factor that promoted hydrate nucleation. As a result, these two factors enhanced the stochastic features of the CO_2_ hydrate nucleation reaction. Even under the same conditions, critical gas concentrations beyond the threshold that hydrates can spontaneously nucleate were not fixed, but they still exhibited linear relations regarding a set temperature. Taking the significant influences of temperature into account, a new nucleation mechanism for CO_2_ hydrates was established based on the potential of the reaction system. Therefore, this study sheds new light when explaining the reason for the formation of gas hydrates in natural reservoirs.

## Introduction

Gas hydrates are one type of ice-like crystal compound, which are comprised of water and gas under suitable temperature and pressure conditions^1^. Water molecules form hydrogen-bonded polyhedral cavities, and small gas molecules are encaged inside^2^. Most components of natural gas can form hydrates, including carbon dioxide, methane, hydrogen and other similar size gases, as well as several low molecular hydrocarbons, such as neo-hexane (NH), tetrahydrofuran (THF) and other hydrocarbons of similar size^3^. Depending on the differences in the gas components, hydrates present different applicable contributions. In recent years, carbon dioxide hydrate has shown great potential in several fields. To meet the CO_2_ emission control demand, CO_2_ capture and storage (CCS) technologies have been considered by researchers using the hydrate-based gas separation (HBGS) process^4–7^. CCS is estimated to be able to contribute more than 17% of the reduction in cumulative CO_2_ emissions using power generation, which would allow the long-term temperature increase to be effectively limited within 2 °C^8^. The HBGS process has been considered a promising method to separate CO_2_ from the CO_2_/CH_4_ mixture in shale gas with a high efficiency^6,9,10^. This process has also been widely used to capture CO_2_ from other mixtures, such as CO_2_/H_2_^4^ and CO_2_/N_2_ flue gas^11,12^. After separation, CO_2_ can be sequestered under the deep sea floor or within natural gas hydrate reservoirs to replace CH_4_^13–16^. In order to enhance crop photosynthesis^17,18^, CO_2_ hydrate has recently been proposed as a replacement for CO_2_ fertilization in the agricultural industry. In addition, methane hydrate is also attractive because of tremendous energy potential. The main component of natural gas is methane and a large number of bore holes in recent decades has demonstrated that natural gas hydrates occur widely on Earth^19,20^. It is estimated that the total amount of carbon stored in the reservoirs of natural gas hydrates is at least twice the total amount of exploitable hydrocarbon reserves stored in traditional fossil fuels^21–23^. Due to these huge reserves, natural gas hydrates also play important roles in global climate change^24^, carbon cycling^25^ and geological disaster prevention^26–28^. Therefore, gas hydrates and some applications related to them have been studied widely.

Different structures of gas hydrates have been confirmed by laboratory experiments^2^. In addition, three-phase equilibrium curves comprised of temperature and pressure conditions have also been established^1^. The driving forces of hydrate decomposition and formation have also been defined with either subcooling or over-pressure relative to the equilibrium curves. The study of hydrate decomposition kinetics is mainly required due to the need to exploit vast methane resources in natural gas hydrate reservoirs^3^. Studying formation kinetics is important for other hydrate technology applications, such as the storage and transport of natural gas, gas mixture separation^29–31^, the replacement of CH_4_ recovery with CO_2_ hydrate formation during the exploitation of natural gas hydrates^14–16^, and CO_2_ capture and sequestration when limiting the increase in future temperatures^32,33^. Using three-phase equilibrium curves, the decomposition of hydrates can be accurately predicted because these equilibrium curves refer to the necessary minimum temperature and pressure conditions for an infinitesimal amount of hydrate to maintain a stable state^34^. By contrast, the nucleation and growth processes of hydrates have not yet been completely understood^35^. As a result, the definitive formation mechanism of gas hydrates has also not been clarified^36^.

To understand the formation mechanisms of hydrates, many experimental methods ranging from macroscopic to microscopic scales have been widely used^37,38^. As mentioned above, subcooling or over-pressure method has been widely applied as the driving force in hydrate formation. With a theoretical model, the chemical potential difference between hydrate crystals and solutions was calculated, and the driving force for hydrate crystallization was finally confirmed to be the concentration of gas^39^. With Raman spectra, it was observed that water molecules in a type-I cage-like structure surround methane molecules in a methane + water solution^40^. Using molecular dynamic (MD) simulations, it was confirmed that gas molecules dissolve in water and form amorphous clusters in water-mediated configurations^41^. These clusters were deemed precursors of hydrate nucleation. With macroscopic measurement methods, including Raman spectroscopy and X-ray diffraction, it was confirmed that the specific formation pathways of clusters were obviously affected by the size and solubility of the gas molecules^42^. Wash *et al*. also used MD simulations and reported that only when the concentration of methane gas in a solution reached a certain critical value did the hydrate begins nucleating^36^. Following this study, Guo and Rodger confirmed that the critical gas concentration that triggers the nucleation of CH_4_ hydrate is 0.05 mole fraction of gas/water^43^, and He *et al*. confirmed that for the nucleation of CO_2_ hydrate, the critical gas concentration is 0.08 mole fraction^44^.

The above findings from MD simulations show that the dissolving property of gas on a macroscopic level during the induction period is a very important factor that dominates the nucleation of hydrates. This property mainly includes the final amount of dissolved gas and the changes in dissolving rates of gas as a function of time. For the final dissolved amounts, the MD simulations provided different conclusions for the two gas categories. However, both conclusions did not include specific formation conditions comprised of temperature and pressure. In addition, changes in the dissolving rates of gas during the whole dissolving process before hydrate nucleation have not been studied in detail. Therefore, we designed a series of experiments on CO_2_ hydrate formations under different temperatures. The dissolving properties of gas during the entire dissolving process (until CO_2_ hydrate nucleation was achieved) were investigated comprehensively.

## Results

### Entire dissolving process of gas before hydrate nucleation

**Figure 1:**
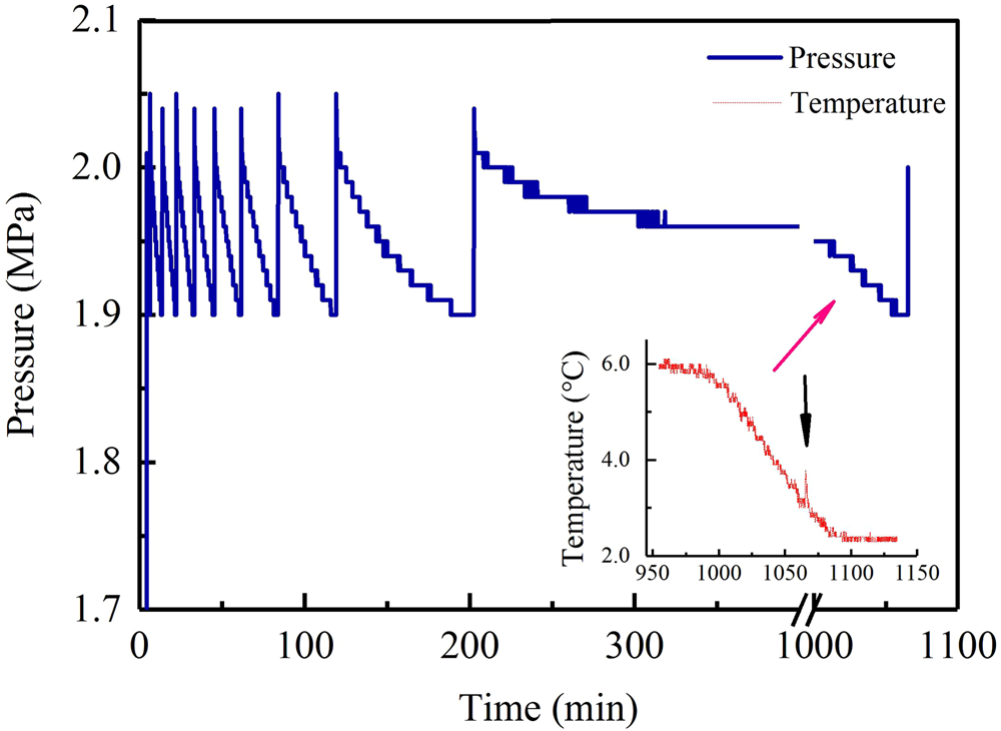
Changes in pressure and temperature as a function of time during the entire gas dissolving process for the first hydrate formation in pure water at 3.5 °C.

In this study, all hydrates were formed by decreasing the temperature at the same rate and in either pure water or an SDS solution with the same volume. As a result, the changing rules of pressure (*P*) and temperature (*T*) during all dissolving processes of gas were generally similar. Figure 1 is representative of the entire process. As shown, due to the instantaneous opening/closing performance of the PID (proportional-integral-derivative) value when retaining constant pressure, the change curve of *P* as a function of time presented a saw-tooth profile. As pressure increased at a constant temperature of 6.0 °C, CO_2_ rapidly dissolved into pure water at the beginning of the experiment. After 200 minutes, the dissolving rate of gas obviously became slow. After 300 minutes, the dissolving process tended to stop, indicating that the solution had been saturated with gas. When the temperature decreased after 980 minutes, CO_2_ dissolved rapidly into the water again, and the solution became supersaturated. When the temperature decreased to 3.1 °C, hydrate crystals precipitated from the solution. At that moment, an obvious increase in the temperature curve occurred due to the heat release from the hydrate nucleation reaction, as shown by the temperature curve in Fig. 1.

**Figure 2:**
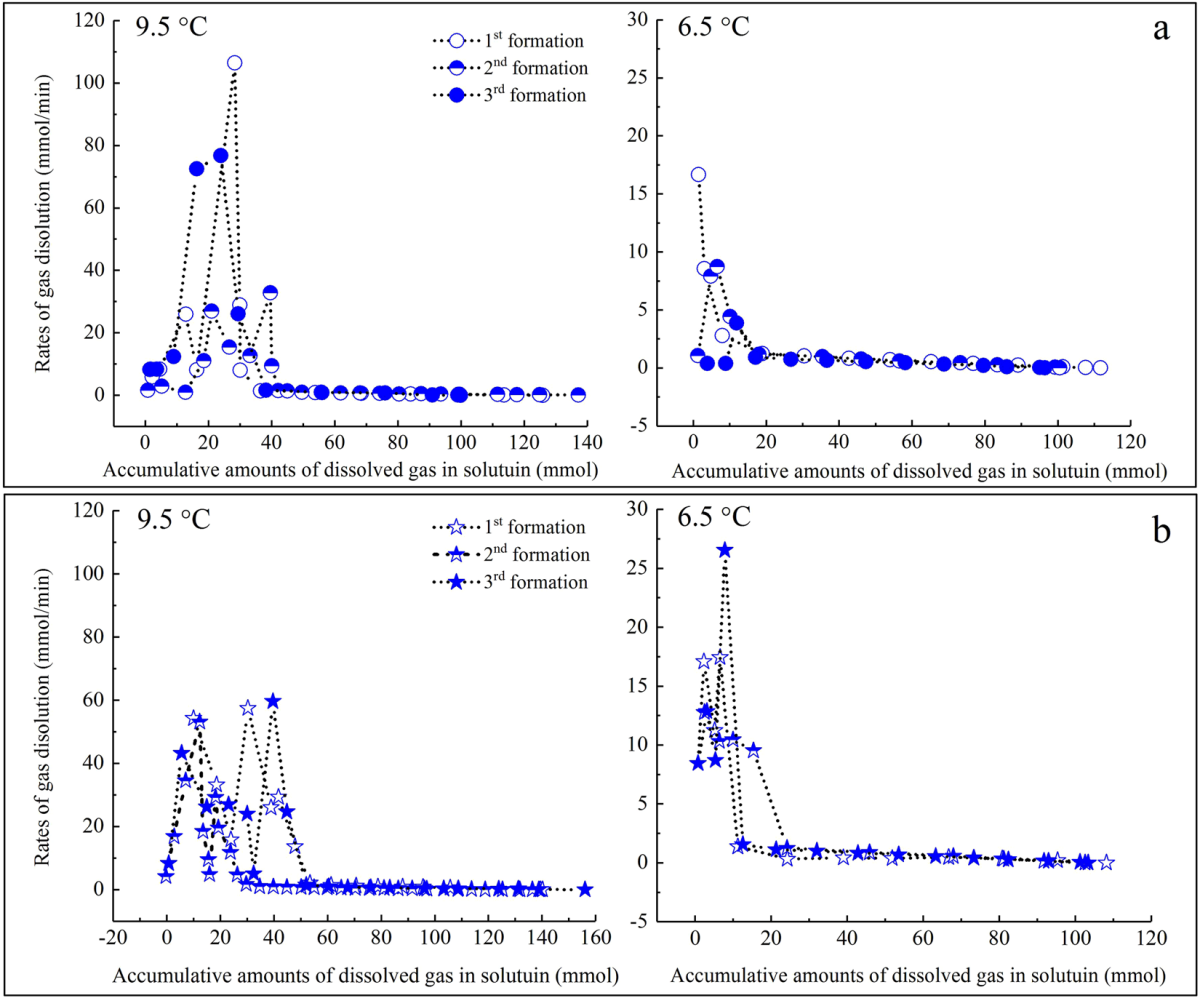
Changes in the dissolving rates of gas as a function of the accumulative amount of dissolved gas during the entire dissolving process for three hydrate formations at 9.5 °C and 6.5 °C. The circle represents gas in pure water, and the star represents gas in the SDS solution.

Each dissolving process before hydrate nucleation consisted of two obvious curve and linear regions (Fig. 2). The curve regions had much higher dissolving rates and smaller amounts of accumulated, dissolved gas. In these rapid dissolving regions, the dissolving rates of gas fluctuated obviously; by contrast, those in the slow dissolving regions decreased monotonously. However, more than 60% of the total amount of dissolved gas was attributed to these slow dissolving processes. The dissolution rules of gas at the other designed temperatures were similar to those shown in Fig. 2.

Combining Figs 1 and 2, it was found that each gas dissolving process before hydrate nucleation was actually in proper order in terms of a fast dissolving region, a slow dissolving region, and a final over-dissolving region.

### Dissolution rules of CO_2_ in different dissolving regions

**Figure 3:**
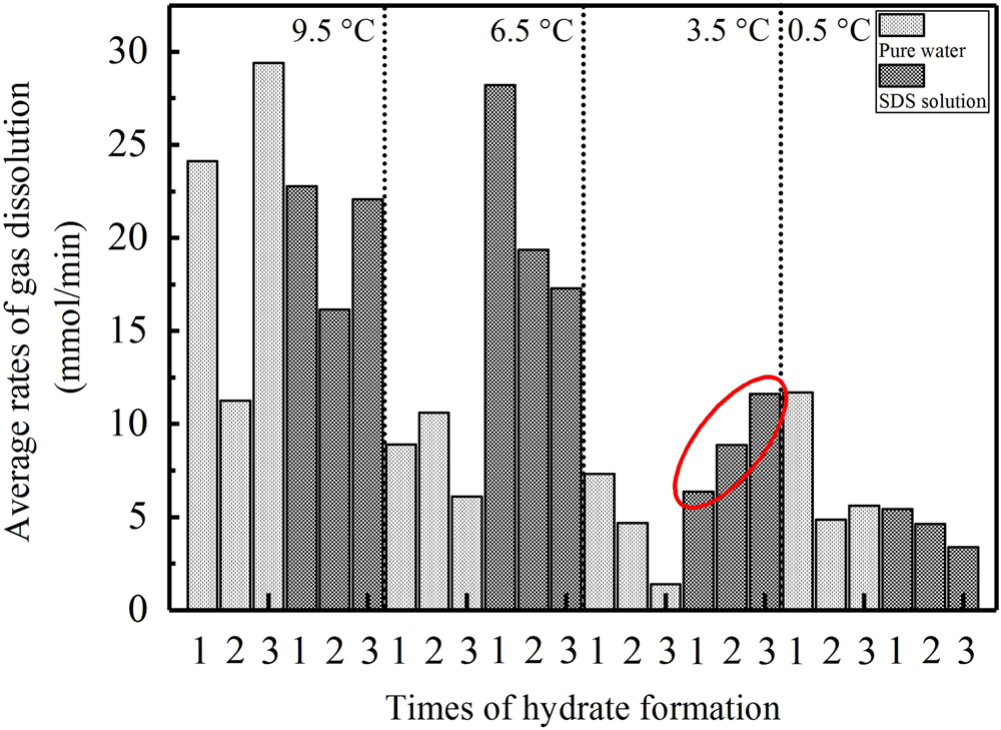
Averages of the dissolving rates of gas in the fast dissolving regions for all experiments.

For the fast dissolving region, the average dissolving rates of gas in each experiment were calculated, and the results are shown in Fig. 3. It can be seen that the change rules for every group of repeated formations did not present obvious monotonous patterns. However, the total averages of the dissolving rates monotonously decreased following the experimental reduction in temperature: 20.96 mmol/min at 9.5 °C, 15.08 mmol/min at 6.5 °C, 6.70 mmol/min at 3.5 °C, and 5.94 mmol/min at 0.5 °C. These total averages represented the differences in dissolving rates under different temperatures; therefore, they were calculated by averaging the sum of the dissolution rates in both water and SDS.

**Figure 4:**
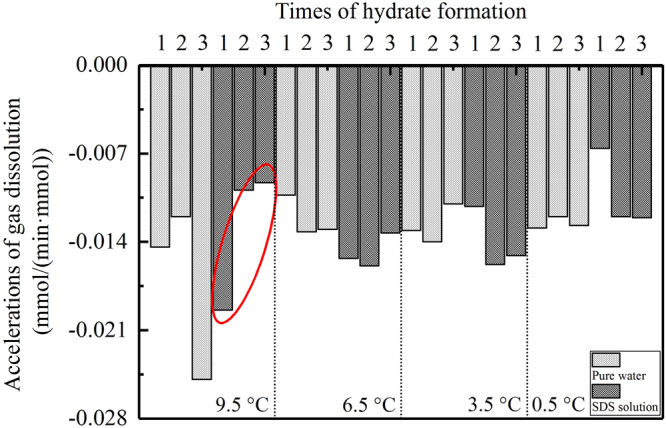
Accelerations of the dissolving rates of gas in the slow dissolving regions for all experiments.

In the slow dissolving region, the dissolving rates differed slightly, and all of them presented obvious linear digressions, as shown in Fig. 2. The dissolving rates were then derived from the accumulative amounts of gas, and the calculated results are shown in Fig. 4. Similar to Fig. 3, the acceleration change rules also presented no obvious monotonous patterns; however, their total averages monotonously increased following a temperature reduction: −0.0149 mmol/(min·mol) at 9.5 °C, −0.0135 mmol/(min·mol) at 6.5 °C, −0.0134 mmol/(min·mol) at 3.5 °C, and −0.0114 mmol/(min·mol) at 0.5 °C.

**Figure 5:**
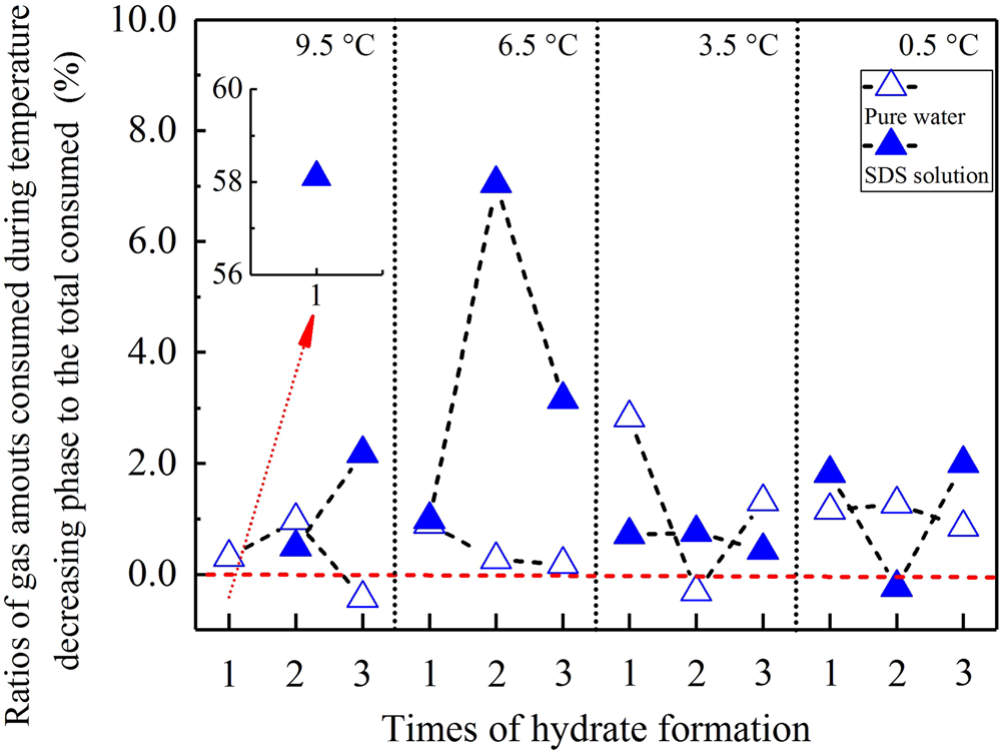
Ratios of the amount of gas dissolved during the temperature reduction phase compared to the total amount of dissolved gas before hydrate nucleation.

**Figure 6:**
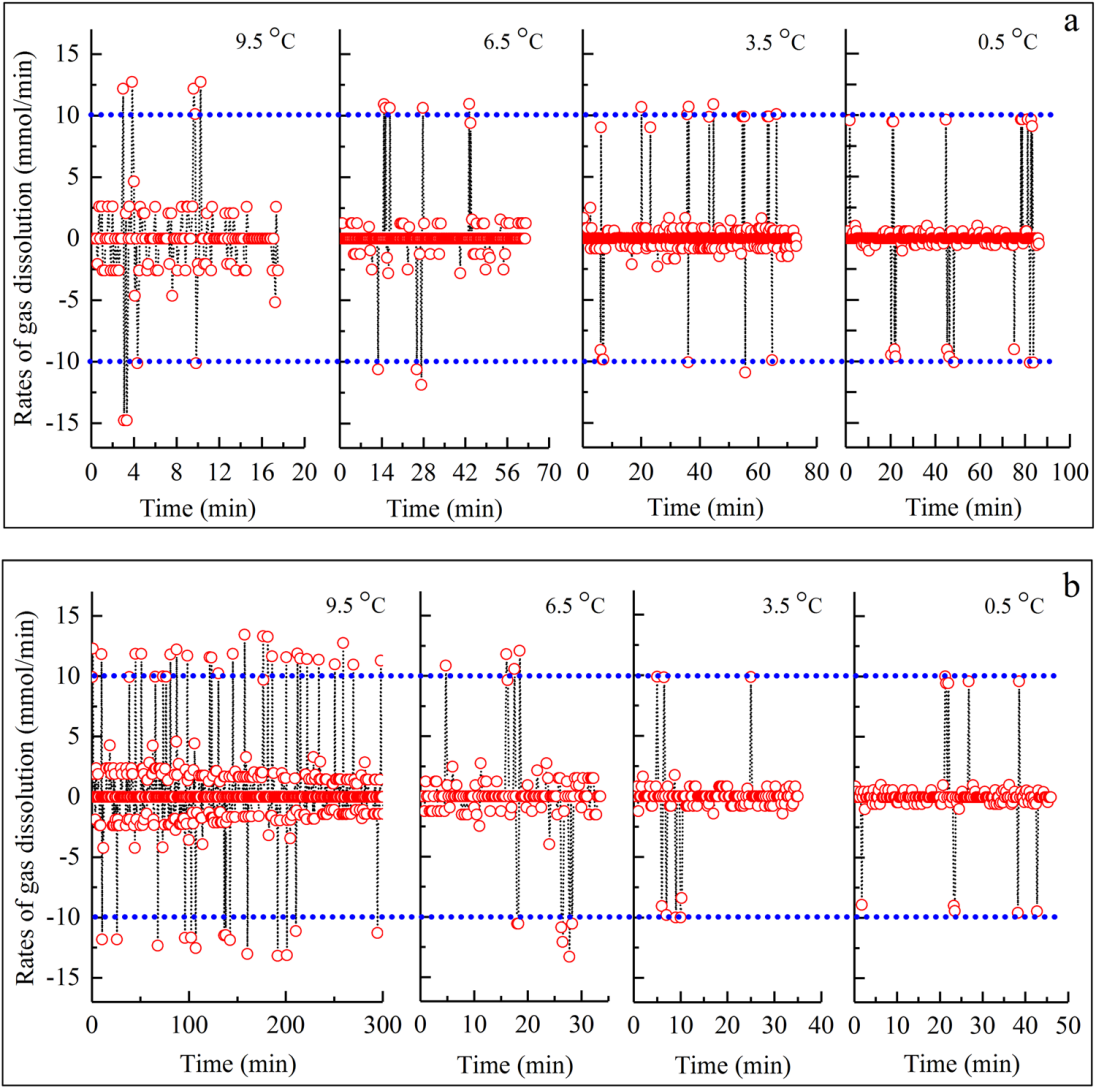
Changes in the dissolving rates of CO_2_ as a function of time during the temperature reduction phase (only for the 1^st^ formation process of the hydrate) at different temperatures. Variable a implies gas in pure water, and variable b implies gas in the SDS solution.

**Figure 7:**
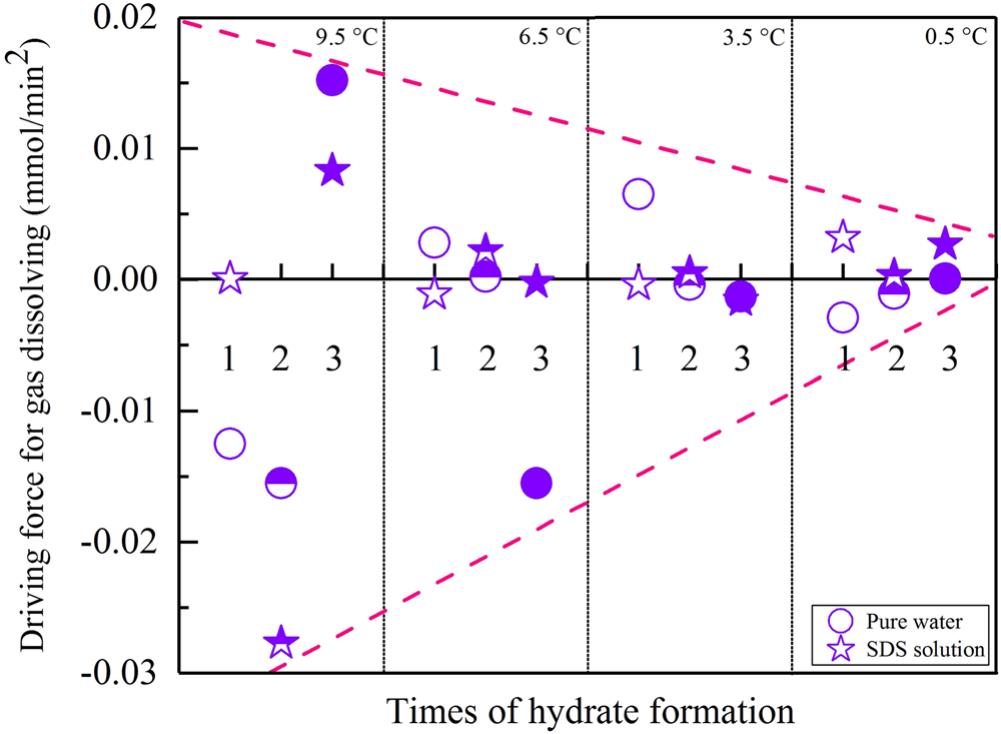
Accelerations in the CO_2_ dissolving rates during the temperature reduction phases in all experiments. The hollow, semi-hollow, and solid symbols imply the 1^st^, 2^nd^, and 3^rd^ formations of the hydrate, respectively.

Following a decrease in temperature, CO_2_ dissolved again, and the solution became supersaturated, as shown in Fig. 1. In addition, only very small amounts of gas were dissolved during this final over-dissolving phase. Figure 5 shows that for most formation processes, the amounts of CO_2_ during the final dissolving phase were smaller than 4.0% of the total amount of dissolved gas during the entire dissolving process. In a total of 24 experiments, only two experiments presented amounts higher than 4.0%: 58.11% for the 1^st^ formation in an SDS solution at 9.5 °C and 7.05% for the 2^nd^ formation, which was also in an SDS solution at 6.5 °C. It should be noted that there were still three negative values for the amount of dissolved gas: −0.43% for the 3^rd^ formation in pure water at 9.5 °C, −0.32% for the 2^nd^ formation in pure water at 3.5 °C, and −0.23% for the 2^nd^ formation in SDS solution at 0.5 °C (as shown by the red dashed lines in Fig. 5). In addition to these dissolved amounts, the dissolving rules of gas during the temperature reduction process were also studied in detailed. As shown in Fig. 6, the dissolving rates vibrated positively and negatively based on time, and the magnitudes presented symmetry. In addition, the vibrating magnitudes gradually decreased following an experimental temperature reduction from 9.5 °C to 0.5 °C (the dashed lines in Fig. 6). It should be noted that only the dissolving rules for CO_2_ during the 1^st^ formation process of hydrates at different temperatures are representatively illustrated in Fig. 6 because the dissolving rules during the other two formation processes were similar. Subsequently, the rules of the detailed gas dissolution accelerations were also investigated. As illustrated in Fig. 7, these accelerations also vibrated positively and negatively, but with different magnitudes. In addition, these vibrating magnitudes obviously presented a gradual decreasing trend following the experimental temperature reduction.

### Total dissolved amounts of gas until hydrate nucleation

**Figure 8:**
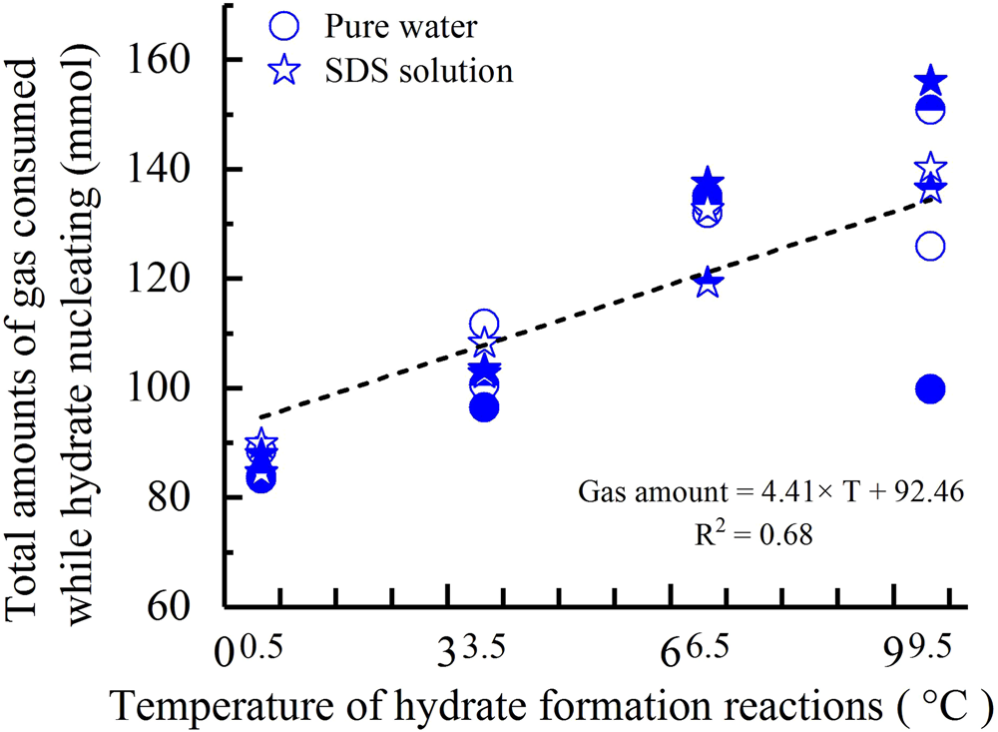
The total dissolved amounts of CO_2_ until hydrate nucleation for all experiments. The calculated results are listed in Table 1.

In addition to the dissolving rules, the total dissolved amounts of gas before hydrate nucleation were also investigated. As shown in Fig. 8, the total amounts of dissolved gas indicated an obvious linear relation with the specific experimental temperatures. Following the increase in temperature, differences in the amount of dissolved gas among the experiments at the same temperatures were more obvious. At 9.5 °C, the difference between the maximum amount and the minimum amount reached 56.24 mmol; one reached 18.28 mmol at 6.5 °C, another reached 15.26 mmol at 3.5 °C, and the last difference reached 6.29 mmol at 0.5 °C. Using the specific data listed in Table 1, all of the average gas concentrations (with a unit of mole fraction of gas/water) before hydrate nucleation under different temperatures were calculated as follows: in the case of pure water, 0.0217 mol/mol at 9.5 °C, 0.0231 mol/mol at 6.5 °C, 0.0178 mol/mol at 3.5 °C, and 0.0148 mol/mol at 0.5 °C; in the SDS solution, 0.0249 mol/mol at 9.5 °C, 0.0224 mol/mol at 6.5 °C, 0.0181 mol/mol at 3.5 °C, and 0.0151 mol/mol at 0.5 °C.

**Table 1:** Properties of the hydrate nucleation reaction and the calculated results for the accelerations of gas dissolution over the linear region in Fig. 2.

Formation temperature	Formation time	Pure water				SDS solution			
		Induction time (s)	Total amount of dissolved gas (mmol)	Slope (mmol/min^2^)	R^2^	Induction time (s)	Total amount of dissolved gas (mmol)	Slope (mmol/min^2^)	R^2^
9.5 °C	1	1050	125.90	−0.0144	0.96	18225	140.18	−0.0194	0.92
	2	840	150.90	−0.0120	0.86	600	136.41	−0.0099	0.98
	3	230	99.83	−0.0249	0.97	1970	156.07	−0.0093	0.95
6.5 °C	1	3735	132.00	−0.0103	0.98	2020	132.61	−0.0153	0.90
	2	2965	133.70	−0.0132	0.99	2965	119.18	−0.0159	0.98
	3	1190	135.10	−0.0130	0.96	4540	137.46	−0.0133	0.98
3.5 °C	1	4385	111.7	−0.0131	0.99	2115	108.20	−0.0112	0.98
	2	1030	100.60	−0.0140	0.99	2135	102.58	−0.0158	1.00
	3	2740	96.44	−0.0110	0.99	1250	103.37	−0.0151	0.97
0.5 °C	1	5185	88.52	−0.0129	0.96	2760	89.79	−0.0066	0.99
	2	5160	83.50	−0.0120	0.99	860	84.51	−0.0120	1.00
	3	3335	83.82	−0.0127	1.00	2505	87.84	−0.0121	0.99

## Discussion

**Figure 9:**
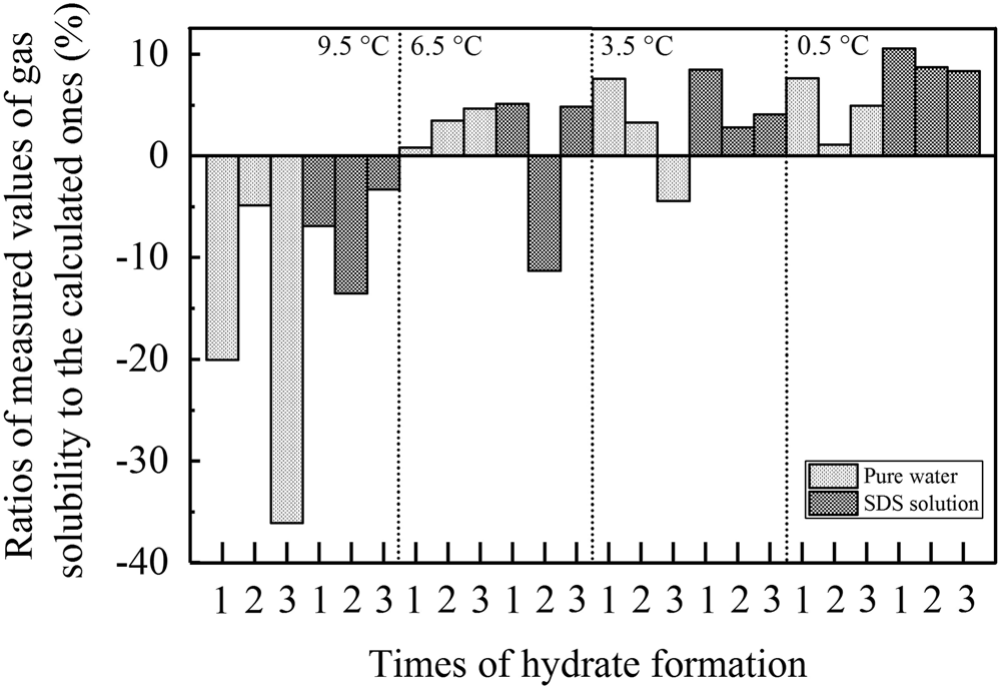
Ratios of the measured amounts of dissolved gas before temperature reduction in each experiment compared to the theoretically calculated amounts of dissolved gas.

To verify the validity of our measurements, all measured amounts of dissolved gas before the decrease in temperature were compared against theoretically calculated values obtained by an online calculation method (http://calc.kl-edi.ac.cn/Default.aspx) established by Duan and Sun^45^. As shown in Fig. 9, while the experimental temperature is ≤6.5 °C, the measured results show excellent consistency with the online calculated results, with a minimum ratio of −11.29% and a maximum ratio of 10.59%. The majority of ratios in Fig. 9 present positive values, and only two ratios have negative values: −11.29% for the 2^nd^ formation of hydrate in the SDS solution at 6.5 °C and −4.43% for the 3^rd^ formation in pure water at 3.5 °C. By contrast, the ratios at 9.5 °C are all negative, and the differences between the measured and calculated results are much larger, ranging from −36.12% to −3.31%. Altogether, our measurement results have excellent validity and are suitable for further discussion. This is especially true at ≤6.5 °C, where two ratios for the 1^st^ formation in pure water at 6.5 °C and the 2^nd^ formation in pure water at 0.5 °C reach 0.83% and 1.10%, respectively. Therefore, we hypothesize that the much larger differences at 9.5 °C are mainly attributed to the specific dissolving behaviours of CO_2_ at high temperatures and not the validity of our measurement method. Because the measurement of gas solubility was performed only through the temperature-retaining period, the above calculation method, which is available under equilibrium conditions, is also available under our experimental conditions. Furthermore, the excellent consistency between the measured and calculated results implies that CO_2_ was finally and thoroughly dissolved in the aqueous phase under stirring conditions in each experiment.

Because the formation process was repeated three times under each temperature condition, the formation mechanism of the ‘memory effect’ of hydrate (i.e., easier hydrate reformation after experiencing dissociation)^46^ can be analysed further using the dissolving rules of gas in different regions, as shown in Fig. 2. It is generally understood that hydrate does not totally decompose during the disassociation process, but it leaves a partial structure; this residual structure enables hydrates to readily form following a second temperature decrease^47^. This viewpoint is mainly supported by several results from macroscopic measurement methods. With MD simulations, an enhanced level of ice and clathrate structures in liquid water during hydrate decomposition was also confirmed^48^. However, based on formation reasons involving the ‘memory effect’ of hydrate, there are still controversies. When measuring neutrons diffracted from tetrabutylammonium chloride in deuterated water with different thermal histories after hydrate dissociation, no significant differences in the total structures among mixtures were found by Benmore *et al*.^49^. In addition, when using neutron diffraction, no significant differences in water structures before and after undergoing the methane hydrate formation process were found by Buchanan *et al*.^50^. Through the investigation on Xe-hydrate crystallization by utilizing synchrotron X-ray computed tomographic microscopy, it was also concluded that the relic structure of water was not the direct cause of the ‘memory effect’ on hydrate^51^.

Lee *et al*. found that with the ‘communication function’ of hydrate formation, the ‘memory effect’ could be propagated to other new water droplets from 5 mm apart if it was made of water resulting from the hydrate decomposition^52^. The experimental results obtained in this study provide further evidence supporting the hypothesis that the ‘memory effect’ of hydrates should not be attributed to residual water structures. If they are, the dissolving ability of gas in a solution should be increased significantly by these residual structures based on the repeated formation of hydrate^53^. However, for the eight experimental groups under different temperatures, only the average dissolving rate of one group (3.5 °C) in the SDS solution in the fast dissolving regions increased monotonously, as shown by the red ellipse in Fig. 3. Similarly, accelerations in the gas dissolution for only one group of experiments (at 9.5 °C in the SDS solution in the slow dissolving regions) increased monotonously, as shown in Fig. 4. In contrast, there were three groups of experiments in Fig. 3 and one group in Fig. 4 which experienced a monotonous decrease in the average gas dissolving rate: 6.5 °C in the SDS solution, 3.5 °C in pure water, 0.5 °C in the SDS solution, and 0.5 °C in the SDS solution, respectively. This means that after experiencing repeated formations, the gas dissolving ability was not enhanced, but it weakened dramatically. Taking into account the strengthening effect of the residual water structures on gas dissolution^53^, we hypothesize that the residual gas molecules in a solution after hydrate dissociation might play a more significant role in the formation of the hydrate ‘memory effect’ compared with the residual water structures. In fact, gas molecules agglomerate due to entopic driving while dissolving in water^54,55^. In addition, using MD simulations, Bagherzadeh *et al*. confirmed that gas molecules can agglomerate and form nano-bubbles in the liquid phase during hydrate dissociation^56^. Due to these nano-bubbles, the gas concentration in a solution can increase, and the dissociation rate of hydrate can slow down^57^. Through experiments on methane/THF hydrates, Veluswamy *et al*. confirmed that numerous spherical-shaped bubbles can steadily exist in the aqueous liquid phase for a long duration of time after hydrate dissociation^58^. With a transmission electron microscopic (TEM), the formation of micro- and nano-bubbles (MNB) in water after C_2_H_6_-hydrate dissociation was identified by Uchida *et al*.^51^. This study also noted that MNBs dominate the memory effect of gas-hydrate crystallization^51^. Altogether, because numerous gas bubbles at the microscopic scale exist in a solution, the dissolving efficiency of gas during the following formation process of a hydrate will be weakened naturally. On the other hand, these microscopic gas bubbles that are dispersed in water have unique properties, such as a negligible increase speed^59^, a higher inner pressure^51^, surface electric charge^60^ and specific surface energy, which break up first when the temperature decreases again. As a result, the local gas concentration in a solution enhances effectively, and the induction time of the hydrate nucleation is shortened dramatically, as listed in Table 1.

After experiencing a fast and slow dissolving process, the solution of CO_2_ becomes saturated, as shown in Fig. 1. Following the decrease in temperature, the solution becomes super-saturated, and the hydrate begins nucleating. In fact, only very small amounts of gas can be dissolved again into a solution that has attained a saturated status during the temperature reduction process, as shown in Fig. 5. This means that the nucleation reaction of the hydrate is actually a type of chain reaction. Only after being triggered by small amounts of dissolved gas, solid hydrate crystals can propagate rapidly throughout the super-saturated solution. In addition to the isotropic thermodynamic property of the liquid phase^61^, this chain characteristic for nucleation reactions is another significant reason for the stochastic feature of hydrate nucleation. Because the amounts of extra dissolved gas, which are required for triggering the nucleation reaction of hydrate, are very small, the specific location where the hydrate crystal nuclei first occur cannot be predicted accurately. Moreover, our experimental results show that during the induction period, the amount of dissolved gas is still not the sole dominant factor for triggering hydrate nucleation. The three negative ratios below the red dashed line in Fig. 5 indicate that several small amounts of CO_2_ dissolve from the solution during the temperature reduction process (in the three experiments at 9.5 °C, 3.5 °C, and 0.5 °C). Therefore, CO_2_ concentrations in the three experiments are reduced following a decrease in temperature. Considering that all experiments were conducted under constant pressures, the sole reason for hydrate nucleation was found to be low temperature. With MD simulations, Guo and Rodger confirmed that during the hydrate formation process, low temperature was conducive for establishing the clathrate structure of water, and high pressure impedes that result^43^. In the abovementioned experiments, although small amounts of gas dissolved, the experimental temperature decreased. As a result, several local gas concentrations were enhanced through the establishment of water clathrate structures due to low temperature. In addition, the nucleation reactions of hydrates were also triggered.

Temperature can also significantly affect the dissolving rates of gas during the temperature reduction process. Under higher temperatures, water molecules have a higher motion energy. As a result, the magnitudes of the gas dissolving rates become higher when the experimental temperature is higher, regardless of whether the solution is pure water or SDS, as shown in Fig. 6. On the other hand, the acceleration amplitudes (in mmol/min^2^) calculated by the gas dissolving rates also clearly attenuate following the decrease in temperature, as shown in Fig. 7.

According to the conclusions from the MD simulations, the gas concentration in a solution is a key factor dominating hydrate nucleation. In addition, it has been confirmed that only when the gas concentration reaches a certain critical value during the induction period does the hydrate begin spontaneously nucleating^36^. For methane and carbon dioxide hydrate, the calculated results by the MD simulations when examining the critical gas solubility that triggers hydrate nucleation were 0.05 and 0.08 mole fraction of gas/water, respectively^43,44^. As shown by the abovementioned calculated results in the ‘Results’ section, our experimental results have the same order of magnitude as those from MD simulations; however, they are still much smaller. The main reason for this is that the stirring function used by our crystallizer improves the dissolving efficiency of CO_2_ significantly. In addition, our experimental results indicate that the critical gas concentrations that trigger hydrate nucleation are not strictly consistent, even for repeated formations under the same conditions. In addition, when the temperature reaches 9.5 °C and 6.5 °C, the critical concentrations begin presenting several stochastic properties, as shown by the date listed in Table 1. As a result, when the experimental temperature is higher, the conclusion is that the critical gas concentration for triggering hydrate nucleation becomes more negative, as shown in Fig. 8. In addition, Fig. 8 also shows that the critical gas concentration tends to rise following an increase in temperature. This means that for studies on the nucleation mechanisms of hydrates, the specific temperature condition that the formation reaction is conducted under should receive more attention. In addition, this conclusion agrees with that drawn from our previous experiments regarding the influence of temperature on methane hydrate formation within a silica gel powder^62^.

**Figure 10:**
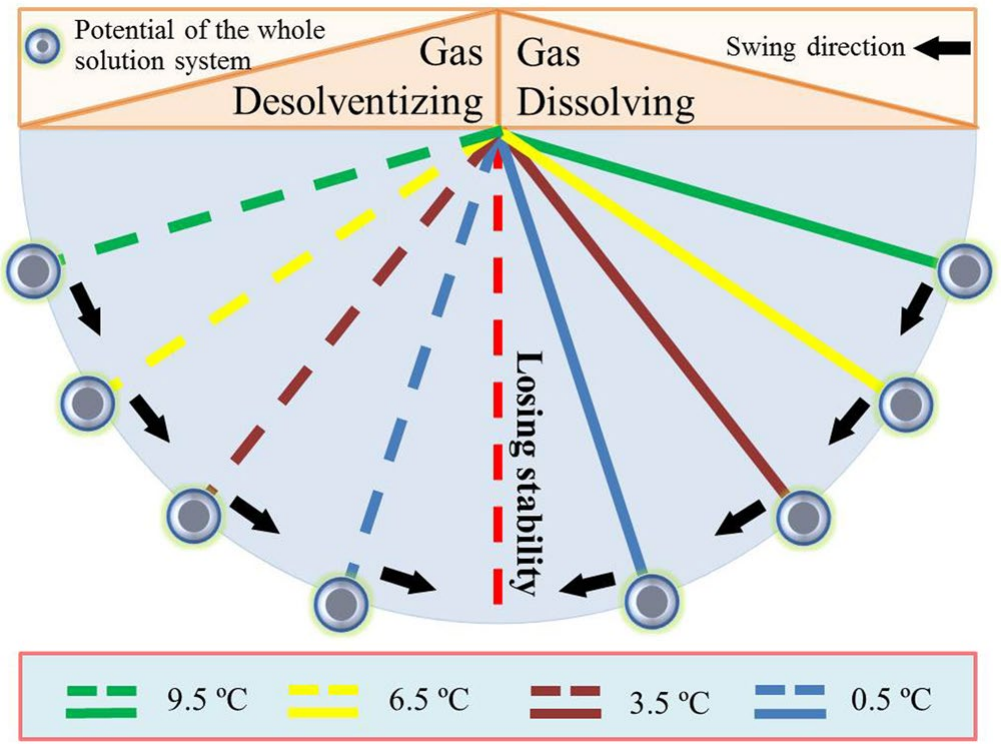
Schematic of the nucleation mechanism for hydrates established based on the potential of a reaction system when considering the influence of temperature on dissolving rates and the final amount of dissolved gas.

Considering the significant influences of temperature on the dissolving rates and final dissolved amounts of CO_2_, we established a new nucleation mechanism for hydrates based on the potential of a reaction system. As illustrated in Fig. 10, when the experimental temperature is higher, the gas and water molecules in the whole solution system have a higher motion energy; which causes the whole system to possess a greater potential during a gas dissolving process that is caused by a decrease in temperature. Then, the whole system swings acutely between the gas dissolving and the desolventizing actions (Fig. 6). As shown in Fig. 6, the amplitudes of the positive and negative gas dissolving rates are generally symmetric; as a result, the swing amplitudes of the solution system potential (illustrated in Fig. 10) are also symmetrical. If the potential of a system is greater, the linear velocity of the small ball (drawn in Fig. 10) will also be higher when it arrives at the lowest point of the circle. In addition, this lowest point has the highest possible position, which can cause the whole system to lose its stability. At this point, the linear velocity of the ball is at its maximum, which causes the exterior force needed to retain the stability of the whole system to also reach its maximum. Figures 6 and 7 show the magnitudes of the dissolving rates and the dissolution accelerations, which are larger under higher temperatures. As shown in Fig. 10, these larger magnitudes imply that a whole solution system with a greater potential is more susceptible to lose stability than that for precipitated hydrate crystals. As a result, the induction period of hydrate nucleation will become short under high experimental temperatures. As shown in Table 1, the average induction periods are 938 s at 9.5 °C (resulting in an abnormal point of 18225 s), 2575 s at 6.5 °C, 2481 s at 3.5 °C and 3460 s at 0.5 °C.

This abnormal point of 18225 s might be mainly attributed to the addition of promoter SDS under a high temperature of 9.5 °C. The specific role of surfactants in promoting hydrate formation is still undetermined^63^. Supposedly, a micelle formation caused by SDS solubility creates several sites for nucleation and enhances the formation rate of hydrates^64^. Similar to the opinion of Uchida *et al*.^51^ regarding the acceleration effect of gas nano-bubbles on hydrate nucleation, we suggest that the promoting function of SDS for hydrate nucleation is mainly attributed to sites at the nanometre level. Under a high temperature of 9.5 °C, these nanosites should agglomerate together, and the sites for nucleation produced by SDS should decrease substantially, causing the induction period of nucleation to be sufficiently prolonged. Due to relic nanosites retained in the solution after hydrate dissociation, the induction periods for hydrate nucleation were shortened substantially during the second and third formation processes of the hydrate, as listed in Table 1.

## Methods

### Experimental apparatus and materials

**Figure 11:**
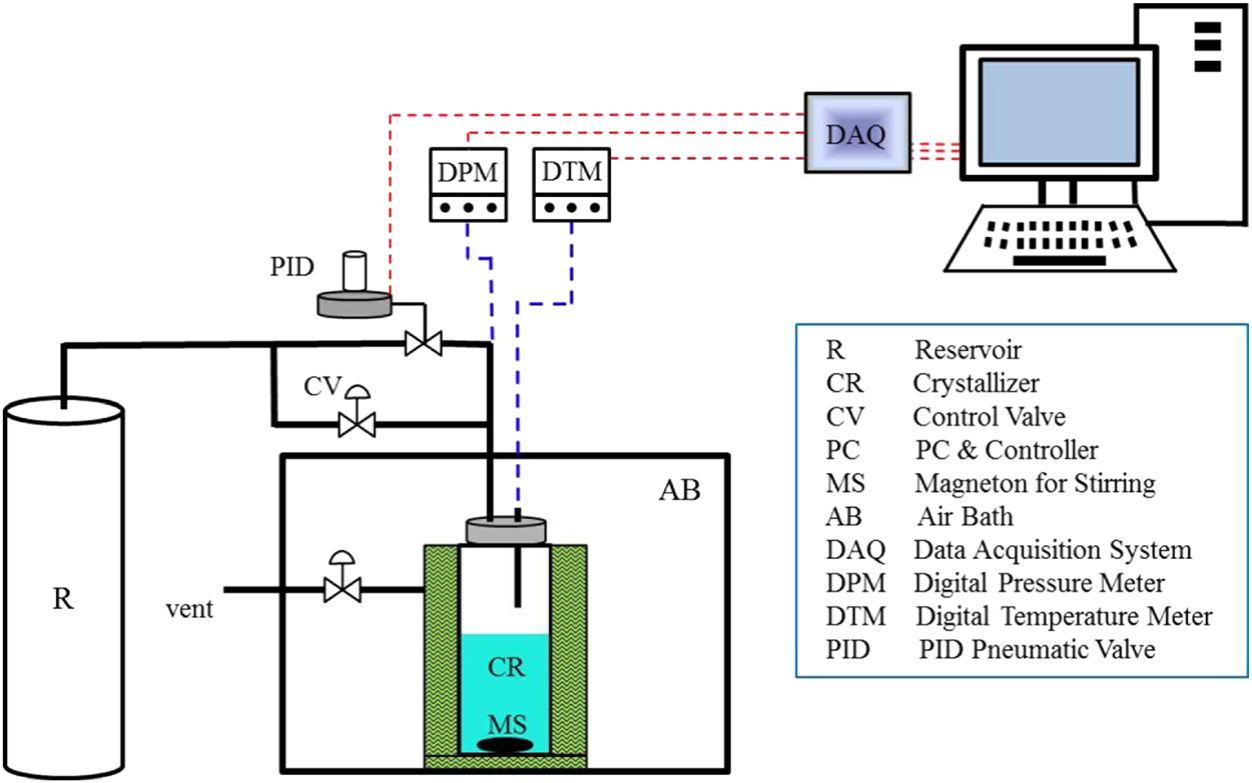
Schematic of the experimental apparatus for hydrate formation.

As shown in Fig. 11, the experimental setup consisted of a cylindrical crystallizer and a data acquisition system (DAQ). The crystallizer was made up of 316 stainless steel, had an internal diameter of 4.5 cm and a height of 13 cm. The crystallizer had a stirring function and was surrounded by a water jacket connected with an external refrigerator. This allowed the temperature of the reactor to be controlled accurately. A pin-type temperature sensor hung from the top base of the crystallizer, with a resolution of 0.1 °C. The pressure and temperature of the experimental system were measured by digital *P* and *T* metres, respectively. The measurement ranges and resolutions of these parameters were −20 to 40 °C and 0.1 °C and 0–15 MPa and 0.01 MPa, respectively. CO_2_ under high pressure was stored in a cylindrical reservoir with an internal volume of 1000 mL, which was also made up of 316 stainless steel. Taking the conduits connected to the crystallizer and reservoir into account, the effective volume of the crystallizer reached 264.72 mL. There was a proportional-integral-derivative (PID) pneumatic valve connected between the crystallizer and the reservoir. During the experiments, all of the measured parameters were recorded and saved by the DAQ at an interval of 5 s. In addition, the PID pneumatic valve was also controlled by the DAQ. The ambient temperature of the experimental system was controlled by an air bath, which was connected with another refrigerator. The crystallizer was connected with a manual injection pump, which allowed either pure water or solution with a certain volume to be injected into the crystallizer before the experiments.

Due to the water-soluble property, CO_2_ with a purity of 99.99% (Yongfang Chemical Co., Ltd., Lanzhou, China) was chosen as the feed gas for hydrate formation. Sodium dodecyl sulphate (SDS) has been widely applied as a promoter of CO_2_ hydrate formations^65–67^. Excluding pure water, CO_2_ hydrates were also formed in SDS solution. As reported, the transfer rate of gas to the aqueous phase was significantly enhanced in the presence of SDS^68^, and a 1 wt% concentration of SDS was very effective in enhancing the rate of hydrate formation^67^. Therefore, an SDS solution with 1 wt% concentration was prepared with analytical grade SDS (Kemiou Chemical Reagent Co., Ltd., Tianjin, China) and deionized water in this study.

### Experimental procedures

Using the temperature reduction method under constant pressure, CO_2_ hydrates formed in pure liquid water and an aqueous solution of SDS. The target temperature conditions of the hydrate formation were designed to be 9.5 °C, 6.5 °C, 3.5 °C, and 0.5 °C. Using the CSMGem software (Natural Gas Hydrate Center, Colorado School of Mines), the equilibrium pressures corresponding to the temperatures were calculated as 4.30 MPa, 2.77 MPa, 1.89 MPa, and 1.32 MPa, respectively. Before each experiment, the deionized water or SDS solution with a volume of 104.1 mL was injected into the crystallizer with a manual injection pump. The magnetic stirrer was then turned on, and the stirring rate was fixed at 200 r/min during each experiment. The ambient temperature of the experimental system was retained at 20 °C using the air bath. The temperature of the crystallizer was set higher than the target temperature by 2.5 °C using a water jacket. Before each experiment, the crystallizer was purged three times with charging/discharging cycles up to 0.5 MPa to expel the residual air inside. After that, the crystallizer was pressurized to the calculated equilibrium *P* value corresponding to the designed *T* in a few steps at an interval of 0.5 MPa and maintained using the PID pneumatic valve. The entire experimental system was then left undisturbed over 12 h under the constant *P* and *T* conditions to allow CO_2_ to sufficiently dissolve in water. After that, the temperature of the crystallizer was uniformly reduced by 4 °C every one hour and twenty minutes, then retained to be constant. During the temperature reduction process, the CO_2_ solution became supersaturated, which precipitated hydrate crystals. To improve the reliability of our experimental results, the above procedures were repeated two times after the 1^st^ formation of the hydrate. As a result, a total of 24 experiments were conducted at four temperatures.

### Calculation methods

The entire CO_2_ dissolving process consisted of many separated segments due to the instantaneous opening/closing performance of the PID valve when retaining a constant pressure. Over each pressurization segment, the amounts and rates of the dissolved gas were respectively calculated using the real gas equation, *PV* = *nZRT*, where *V* represents the volume of the space over the pure water or SDS solution in the crystallizer. In addition, the calculated result for *V* was 160.62 mL, which was the total internal volume of the crystallizer (264.72 mL) minus that of the injected liquid (104.1 mL). The compressibility factor, *Z*, was calculated with the Pitzer correlation^69,70^. To improve the accuracy of the calculated results, *Z* was calculated separately at the beginning and end of each pressurization segment. The total amount of gas during each dissolving process was finally obtained by summing up the total amount of gas dissolved over each segment.

With the dissolving rates of gas, the acceleration of each dissolving process was easily obtained using Microsoft Excel, with units of mmol/min^2^ or mmol/(min·mmol). The former indicates the first derivative of the dissolving rate with time, and the latter indicates the accumulated and dissolved amount of gas.

The elapsed time between the start points of the temperature decrease and the hydrate nucleation was calculated as the induction time before nucleation.

## Conclusions

With a decrease in temperature, CO_2_ hydrates were formed in pure water and an SDS solution under a stirring condition, different temperatures and constant pressures. The dissolving properties of CO_2_ throughout the whole induction period were investigated in detail. The experimental results show that the whole dissolving process of gas before a decrease in temperature can be divided into fast and slow regions. With specific dissolving rates and dissolution accelerations calculated over the two regions, it was confirmed that the ‘memory effect’ of the hydrate was not attributed to the residual water structures after the hydrate dissociation. This was consistent with the directly measured results from the MNB formation in water after C_2_H_6_-hydrate dissociation using the TEM. Altogether, more detailed studies on the reason for the occurrence of the ‘memory effect’ of hydrates in the future should focus on relic gas molecules in the aqueous phase after hydrate dissociation. Because the ratios of consumed gas amounts only occurred during the temperature reduction period compared to those over the whole induction period, CO_2_ hydrate nucleation was confirmed as a type of chain reaction. This enhances the stochastic features of hydrate nucleation. A specific temperature under which hydrate can be formed is another important factor that enhances the stochastic features of CO_2_ hydrate nucleation. Different from the MD simulation results, we suggest that critical gas concentrations beyond which the hydrate begins spontaneously nucleating should not be fixed, even under the same conditions; however, they do present an obvious linear relation to the specific experimental temperature. Finally, considering the significant influences of temperature, we established a new nucleation mechanism for CO_2_ hydrates from based on the potential of a reaction system. This might provide new light when explaining hydrate formation processes in natural reservoirs.
